# Progression of motor neuron disease is accelerated and the ability to recover is compromised with advanced age in rNLS8 mice

**DOI:** 10.1186/s40478-016-0377-5

**Published:** 2016-09-29

**Authors:** Krista J. Spiller, Clark R. Restrepo, Tahiyana Khan, Anna M. Stieber, Linda K. Kwong, John Q. Trojanowski, Virginia M.-Y. Lee

**Affiliations:** Department of Pathology and Laboratory Medicine, Center for Neurodegenerative Disease Research (CNDR), Institute on Aging, Perelman School of Medicine, University of Pennsylvania, Maloney Building, 3rd Floor 3600 Spruce Street, Philadelphia, PA 19104-2676 USA

**Keywords:** Amyotrophic lateral sclerosis, Motor neuron, Reinnervation, rNLS mice, TDP-43, Neuromuscular junction

## Abstract

In order to treat progressive paralysis in ALS patients, it is critical to develop a mouse that closely models human ALS in both pathology and also in the timing of these events. We have recently generated new TDP-43 bigenic mice (called rNLS8) with doxycycline (Dox)-suppressible expression of human TDP-43 (hTDP-43) harboring a defective nuclear localization signal (hTDP-43∆NLS) under the control of the *NEFH* promoter. Our previous studies characterized the pathology and disease course in young rNLS8 mice following induction of neuronal hTDP-43ΔNLS. We now seek to examine if the order and timing of pathologic events are changed in aged mice. We found that the expression of hTDP-43∆NLS in 12+ month old mice did not accelerate the appearance of neuromuscular abnormalities or motor neuron (MN) death in the lumbar spinal cord (SC), though disease progression was accelerated. However, following suppression of the transgene, important differences between young and aged rNLS8 mice emerged in functional motor recovery. We found that recovery was incomplete in aged mice relative to their younger treatment matched counterparts based on gross behavioral measures and physiological recordings from the animals’ gastrocnemius (GC) muscles, despite muscle reinnervation by surviving MNs. This is likely because the reinnervation most often only resulted in partial nerve and endplate connections and the muscle’s junctional folds were much more disorganized in aged rNLS8 mice. We believe that these studies will be an important basis for the future design and evaluation of therapies designed to slow denervation and promote re-innervation in adult ALS patients.

## Introduction

Most patients who develop ALS are between 40 and 70 years old, with juvenile onset occurring only very rarely [20]. Therefore, aging is a risk factor for ALS as well as many other neurodegenerative diseases, and the changes that take place in ALS typically occur on the background of normal aging, in which a complex series of senescent changes take place at every level from molecular to cellular to whole organ systems [29]. In humans, the loss of muscle mass and accompanying degeneration of neuromuscular junctions (NMJs) is called sarcopenia and is a major cause of disability in the elderly [25]. In mice, it has been reported that with advancing age, pre-terminal portions of motor axons exhibit regions of abnormal thinning, distension, and sprouting, whereas postsynaptic endplates decrease in size and become reduced in number, length, and density of postsynaptic folds [12]. In rats, the number of acetylcholine receptors per NMJ are significantly lower in old versus young animals [7]*,* and nerve conduction velocities are significantly diminished [31]. Though the majority of changes have not yet occurred by the median age of ALS diagnosis (54–61 years old depending on the population), it is important to consider these ongoing processes, particularly with regard to long-term disease management.

Despite this, the vast majority of animal modeling for ALS has been done on very young animals. Although mice sexually mature fairly rapidly (by 35 days after birth), maturational growth continues for most biological processes and structures until about 3 months [10]. It is not until beyond 6 months that mice begin to exhibit some age-related changes, and mice are not considered to be “middle-aged” until they are at least 10 months old [10]. Ideally, ALS-relevant mouse-modeling experiments should therefore begin after mature adulthood is reached, i.e. when the mice are middle-aged: around 10 months old.

We recently developed a new mouse model called rNLS8, based on the pathological protein found in over 90 % of ALS patients: TDP-43 [32]. We found that expression of hTDP-43∆NLS resulted in the accumulation of abundant insoluble, phosphorylated TDP-43 in brain and spinal cord, concomitant with nuclear clearance of endogenous mouse TDP-43 (mTDP-43) [32]. Our initial characterization of the rNLS8 mice occurred in animals in which the transgene was expressed starting at the very beginning of their fertility, when they were only “teenagers” at 5 weeks (wks) old. Despite their youth, we still found striking ALS-relevant phenotypes. When NMJs in whole hindlimb muscles were examined, we found significant denervation occurred after only 4 wks of transgene expression in these young rNLS8 mice and the number of motor neurons (MNs) in the lumbar level of the spinal cord decreased by ~28 % after 6 wks of transgene expression [27]. In this study, we now evaluate if the onset and/or disease progression in aged rNLS8 mice are changed compared to younger animals. Previous studies that have examined the effect of the timing of transgene expression have focused more on the marked brain atrophy that is induced by human TDP-43 overexpression during brain development [6] or the neurodegeneration that results from mutant TDP-43 expression in animals post-weaning versus young adults (<6 months old) [1]. We will add to the field by aging mice for approximately 1 year prior to transgene induction.

Indeed, aging should be taken into account in therapeutic strategies designed to increase the likelihood of successful clinical translation. However, as a proof-of-concept, we have used young mice to investigate whether the clearance of TDP-43 pathology is a clinically relevant future therapeutic direction [27, 32]. After 6 weeks of transgene expression, when significant TDP-43 pathology was present with overt neuron loss and dramatic motor decline, we suppressed hTDP-43∆NLS expression and found that the rNLS8 mice showed a rapid functional improvement with a reversal of the hindlimb clasping phenotype and rapid weight gain [32]. Despite the significant muscle denervation and even with the ~28 % reduction in the total number of MNs at the TA-innervation level of the spinal cord, the remaining MNs were able to significantly re-innervate the vacated TA motor endplates following suppression of hTDP-43∆NLS expression [27]. This dramatic recovery is promising, but we now need to assess whether recovery is still possible in older animals. Specifically, it is critical to test whether aged MNs still have the ability to efficiently clear TDP-43 pathology, and if the axonal projections that remain intact can expand to cover the vacated motor endplates by collateral sprouting. Finally, if re-innervation does occur, we also aim to assess whether the newly formed NMJs are functional.

## Materials and methods

### Tg and nTg mice

As described in Walker et al., 2015 [32], rNLS8 mice were generated by crossing new Tg lines overexpressing tetracycline transactivator (tTA) protein under the control of the human *NEFH* promoter with an existing Tg line that can be induced with Dox manipulation to express human TDP-43 with a defective nuclear localization signal (hTDP-43ΔNLS but hereafter refer to as hTDP-43). A Dox diet inhibits tTA from binding to the tetracycline promoter element, repressing hTDP-43 expression. When mice are taken off Dox, hTDP-43 expression is activated. For the cycling experiment, we repeatedly expressed and suppressed the hTDP- 43ΔNLS transgene for 2 wks stretches over the course of 3 months or 12 months and then removed Dox continuously for 4 wks prior to sacrifice. These mice were sacrificed at various time-points, including disease end-stage, which was defined as 30 % weight loss from peak. For all studies, nTg littermates from the biogenic cross are used as controls and both male and female mice were used.

### Study approval

All procedures were performed in accordance with the NIH Guide for the Care and Use of Experimental Animals. Studies were approved by the Institutional Animal Care and Use Committee of the University of Pennsylvania.

### Electron microscopy

Sciatic nerve and tibialis anterior muscles were fixed by perfusion and 2–4 days immersion in 2.5 % glutaraldehyde + 1 % paraformaldehyde in 0.1 M cacodylate buffer, pH 7.4 + 0.005 % CaCl_2_, washed, dehydrated in ethanol, and embedded in epoxy resin (EMbed 812, Electron Microscopy Sciences, Hatfield, PA). One μm cross-sections were stained with toluidine blue for light microscopy and areas with neuromuscular endings were selected for ultrathin sectioning, cut, and stained with 2 % uranyl acetate in 50 % ethanol and Reynold's lead citrate buffer [8].

### Electrophysiological measurements

Similar to previous studies [27], mice were anesthetized and two electrodes were placed on either side of the sciatic nerve at a paraspinal site. Bipolar electrodes were inserted into the GC muscle. A controlled stimulation was applied to the nerve to evoke contractions from the GC muscle in 5 mA increments from 10 mA.

### Succinate dehydrogenase activity stain

Fresh frozen muscle sections were cut transversely at 10 µm and then were incubated for 30 min in a medium consisting of equal parts dH_2_0, nitro blue tetrazolium solution, sodium succinate solution, and 0.2 M phosphate buffer. Sections were rinsed in PBS and fixed in 10 % formalin for 5 min. Sections were then rinsed in 15 % ethanol for 5 min and coverslipped with mounting medium.

### Immuofluorescence and quantification

Tg rNLS8 mice and nTg controls were perfused with ice-cold PBS followed by 10 % formalin and then lumbar spinal cord and hindlimb muscles were surgically removed. Muscles were washed in PBS overnight, and the central nervous system tissue was post-fixed in 10 % formalin overnight. All were then washed in PBS and then processed in a sucrose gradient for cryoprotective embedding.

To visualize NMJs, 30 μm longitudinal cryosections were incubated with α-bungarotoxin conjugated to Alexa Fluor 488 (1:500; Invitrogen) and an antibody to vesicular acetylcholine transporter (VAChT; raised in rabbit or guinea pig, 1:32,000; [27]) to label motor endplates and nerve terminals, respectively. A lack of co-localization indicated muscle denervation.

To analyze MN populations in lumbar spinal cord, the following primary antibodies were used: guinea pig anti-VAChT (1:10,000,[27]); mouse anti-human TDP-43 monoclonal antibody (MAb) (0.06 μg/mL, clone 5104, [27]), and rabbit anti-C-terminal TDP-43 polyclonal antibody (0.15 μg/mL, [27] C1039). After overnight incubation with primaries, sections were washed and then incubated with goat anti-mouse, anti-rabbit or anti-guinea pig 488- or 594-conjugated Alexa Fluor secondary antibodies (1:1000, Molecular Probes). To quench autofluorescence of aged tissue, slides were treated for 10 min with 0.5 mM copper sulfate buffer (50 mM ammonium acetate buffer, pH 5.0), then rinsed with dH_2_O, prior to coverslipping with Vectashield mounting medium. For the quantification of motor neuron numbers, motor neurons were counted in consecutive transverse 20 μm cryosections, as has been previously described [14].

### Preparation of mouse spinal cord lysates

As has been previously described [32], whole spinal cord was dissected on ice and then sonicated in 5 vol (v/w) RIPA buffer (50 mM Tris, 150 mM NaCl, 1 % NP-40, 5 mM EDTA, 0.5 % sodium deoxycholate, and 0.1 % SDS, pH 8.0) containing 1 mM PMSF and protease and phosphatase inhibitor cocktails (Sigma). Samples were centrifuged at 4 °C, 100,000 *g* for 30 min. Protein concentrations of the RIPA-soluble fractions were determined using the bicinchoninic acid protein assay (Pierce).

### Immunoblotting

20 μg of protein were separated by 10 % SDS-PAGE and transferred to nitrocellulose membrane (Bio-Rad). Antibodies used for immunoblotting were 5104 (1:1000), 2340–181 (1:1000; [18]), and a mouse anti-GAPDH MAb (1:5000, clone 6C5, Advanced Immunochemical). Signals were detected using goat anti-mouse or anti-rabbit IRDye-680 or IRDye-800 conjugated secondary PAbs (1:20,000, Li-Cor or Rockland) and imaged using a Li-Cor Odyssey imaging system. Band infrared fluorescent signals were quantified using Li-Cor Image Studio Version 2.0.

### Statistics

Statistical significance was determined using unpaired two tail t-tests when comparing two groups and one-way ANOVA when comparing multiple groups, using SigmaPlot. A *p*-value of less than 0.05 was considered significant.

## Results

### Aging accelerates progression but not the onset of motor neuron disease

**Fig. 1:**
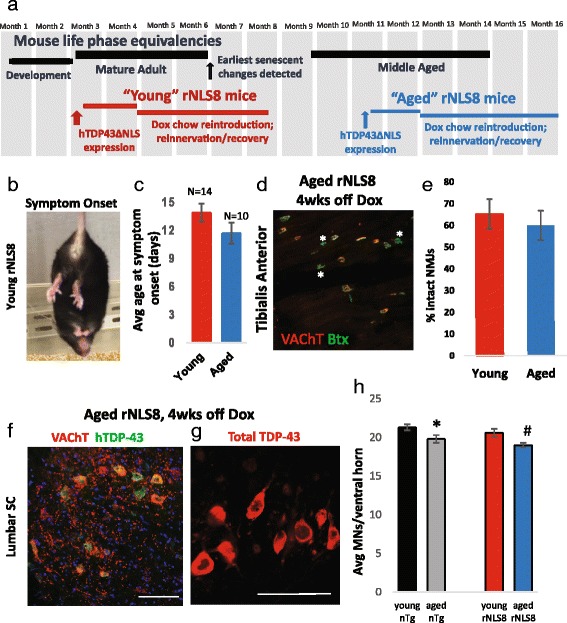
Aging does not affect ALS-like MN disease onset in rNLS8 mice. **a** Experimental timeline with life phase equivalencies indicated. **b** Representative image of a young rNLS8 mouse at symptom onset (notice hindlimb clasping). **c** Aged rNLS8 mice do not show a difference in onset of symptoms compared to young mice as assessed by appearance of one or more of the following phenotypes: clasping of hindlimbs, tremor, weight loss, and/or hunched posture. Mean ± SEM, *p* = 0.14. **d** Representative muscle cryosection from the TA of an aged rNLS8 mouse at 4 wks off Dox, with overlap of VAChT positive motor terminals (*red*) and acetylcholine receptors (α-bungarotoxin, BTX, *green*) as an indicator of innervated motor endplates. Denervated NMJs are marked with white asterisks. Scale bar = 100 μm. **e** After 4 wks of transgene expression, aged Tg mice show no difference in TA innervation when compared to young Tg mice. Data are mean ± SD (*n* = 3–4 for both young and aged Tg mice; *p* = 0.38). **f** Representative immunostaining for VAChT (*red*, to label MNs) and hTDP-43 (*green*) on a cryosection of lumbar SC from an aged rNLS8 mouse at 4 weeks off Dox shows that the majority of MNs express the transgene. **g** When staining with a TDP-43 antibody that labels both hTDP-43 and endogenous mTDP-43, widespread nuclear clearance is observed at this 4 wks off Dox time-point, similar to young rNLS8 SC [32]. Scale bar = 100 μm. **h** A slight decrease is observed in the number of lumbar SC MNs in young versus aged nTg mice (compare *black* vs *grey bar*, *p* = 0.05), but this MN loss is not exacerbated by 4 wks of transgene expression in rNLS8 animals (compare *grey* vs. *blue bar*, *p* = 0.1). ^∗^ *p* = 0.05, compared to young nTg; # *p* < 0.05, compared to young rNLS8 mice

The major advantage of the newly described rNLS8 mouse line is that the hTDP-43∆NLS transgene is inducible by removing doxycycline (Dox) from the animals’ diet and consequently suppressed by the reintroduction of Dox. Thus, we exploited this feature to model ALS and recovery in middle-aged mice to see if the disease course was changed due to aging. We first compared young adult rNLS8 (transgene induced by Dox removal at 3 months) to aged rNLS8 mice (transgene induced by Dox removal between 10 and 12 months) (Fig. 1a). We defined symptom onset as the appearance of any of the following: clasping of hindlimbs, tremor, weight loss, and/or hunched posture (Fig. 1b). Aged rNLS8 mice did not show a difference in the timing of the onset of any of these symptoms compared to young mice, with the earliest symptoms appearing after about 2 wks of transgene expression (*p* = 0.14, Fig. 1c). Moreover, there was not an exacerbation of axonal dieback from the vulnerable, fast tibialis anterior (TA) muscle by 4 wks off Dox, with young and aged mice having lost 35 and 40 % of intact NMJs, respectively (*p* = 0.38, Fig. 1d-e). At the lumbar level of the SC, aged mice showed the same level of transgene expression that young rNLS8 mice with ~76 % of L3-L5 MNs expressing hTDP-43 (Fig. 1f). This cytoplasmic TDP-43 expression resulted in nuclear clearance of endogenous mouse TDP43, similar to young rNLS8 SC [32] (Fig. 1g). Finally, a slight, but statistically significant decrease was observed in the number of lumbar SC MNs in young versus aged non-transgenic (nTg) mice from 21.3 ± 0.4 to 19.8 ± 0.5 avg MNs/ventral horn, respectively (*p* = 0.05), but this MN loss was not exacerbated by 4 wks of transgene expression in rNLS8 animals (Fig. 1h, and also see Fig. 4f). Therefore, aging does not affect motor neuron disease onset.

**Fig. 2:**
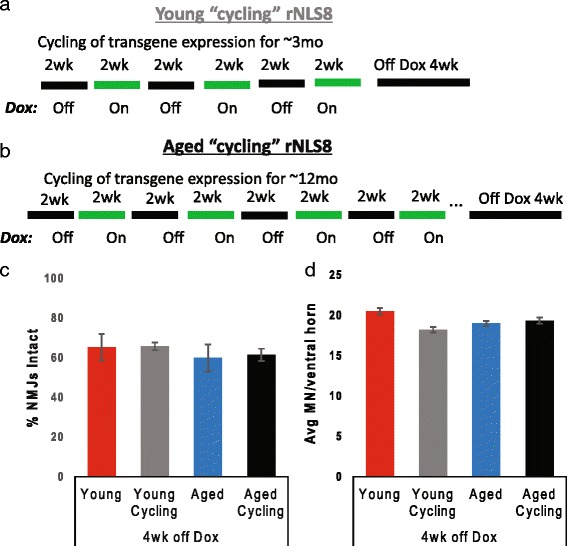
Cell stress does not accelerate disease onset. **a**-**b** Experimental scheme in which rNLS8 animals were weaned and then exposed to repeated cycles of hTDP-43ΔNLS expression (for 2 wks) followed by suppression (for 2 wks) by feeding them chow with or without Dox. This lasted for 3 months in the “young cycling” group (**a**), or 12 months in the “aged cycling” group (**b**) prior to removing Dox continuously for 4 wks. **c**-**d** Repeated transgene expression and suppression, designated “cycling”, for either 3 months (*grey bar*) or for a whole year (*black bar*) does not exacerbate TA denervation (**c**) or lumbar MN loss (**d**) at 4 weeks off Dox compared, to young or aged mice that are off Dox without cycling (*red* and *blue bars*). Data are mean ± SD (*n* = 3–4 for all groups of mice)

Aging is a potent modifier of protein homeostasis [21] and the consequential changes in proteasome activity and oxidative stress have been causally linked to neuronal death in the rodent SC [16]. Given that 10–12 months of aging prior to transgene expression did not alter the appearance of neuromuscular abnormalities in rNLS8 mice, we next asked if we could induce changes by further stressing the motor system through repeated alterations of protein homeostasis. Specifically, we repeatedly changed TDP-43 cellular localization and, consequently, RNA metabolism and processing. To do this, we repeatedly expressed and suppressed the hTDP-43ΔNLS transgene for 2 wks stretches over the course of 3 months (to age-match to “young” rNLS8 mice) or 12 months (to age-match to “aged” rNLS8 mice) and then removed Dox continuously for 4 wks (Fig. 2a-b). We chose these 2 wks timespans based on earlier results that showed high hTDP-43 expression after 2 wks off Dox, and effective clearance after 2 wks on Dox [32]. Surprisingly, this repeated transgene expression and suppression, designated “cycling”, did not exacerbate TA denervation (Fig. 2c) or lumbar MN loss (Fig. 2d) at 4 wks off Dox. Therefore, chronic basal cell stress did not accelerate disease onset.

**Fig. 3:**
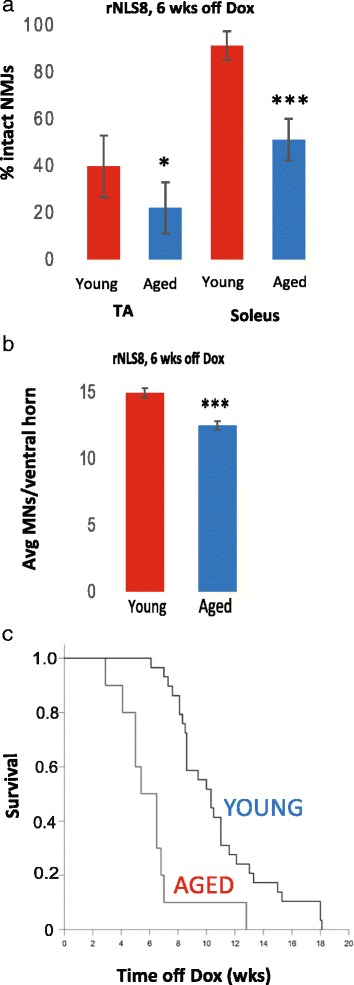
Differences emerge between young and aged rNLS8 mice as the ALS-like disease progresses. **a** After 6 weeks of transgene expression, aged mice show differences in both TA and soleus innervation compared to young mice, *p* = 0.04 and *p* = 8.3E-4, *n* = 3–4. **b** Aged mice also have fewer lumbar MNs than young rNLS8 mice at 6 weeks off Dox. Data are mean ± SD (*n* = 3–4 for both young and aged mice; *p* = 1.2E-04), ^∗^ *p* < 0.05, ***p < 0.001. **c** Aging significantly accelerates time to death after transgene induction in rNLS8 mice. Median time to death is 6.5 weeks for aged mice vs. 10.3 weeks for young mice. (*n* = 10 for aged mice, *n* = 29 for young mice, Log rank test statistic = 18.7, *p* < 0.001)

Though onset was unchanged, when aged rNLS8 animals did get sick, they had a more rapid decline than their younger counterparts. After 6 wks of transgene expression, aged mice show differences in innervation of both TA and the typically more disease-resistant soleus muscle compared to young rNLS8 mice, *p* = 0.04 and *p* = 8.3E-4 (Fig. 3a). Aged mice also have fewer lumbar MNs than young rNLS8 mice at 6 wks off Dox (*p* = 1.2E-04, Fig. 3b). Finally, aging significantly accelerated time to death after transgene induction in rNLS8 mice, with a median decrease in lifespan of about 3.5 weeks in aged mice compared to young (Log rank test statistic = 18.7, *p* < 0.001, Fig. 3c). Therefore, aging accelerated disease progression in rNLS8 mice.

### Motor recovery is diminished in aged rNLS8 mice because of impairments at the NMJ

**Fig. 4:**
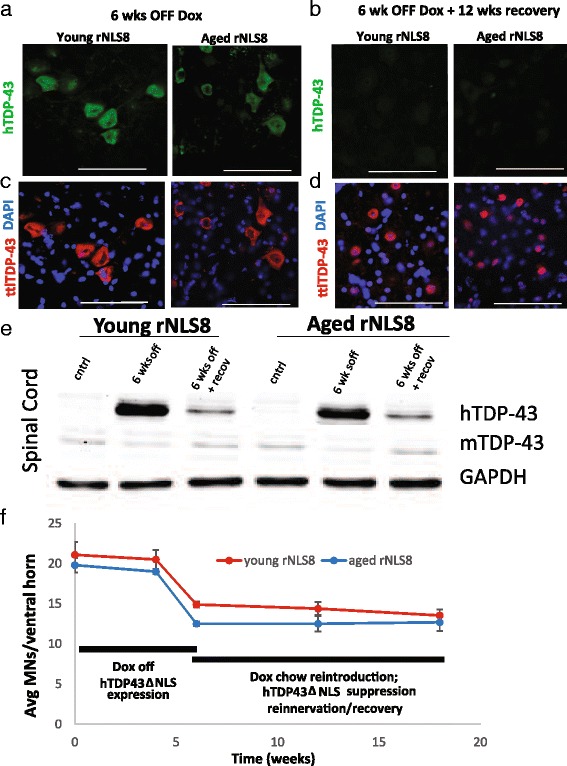
Suppressing the hTDP-43ΔNLS transgene restores endogenous mTDP-43 and halts MN loss in both young and aged rNLS8 mice. **a** Representative pictures of lumbar SC of young (*left*) and aged (*right*) rNLS8 mice at 6 wks off Dox show high, cytoplasmic expression of hTDP-43 (*green*). **b** After 12 wks back on Dox, both young and aged rNLS8 mice show a clearance of this cytoplasmic hTDP-43. **c** When the same cryosections were stained with a TDP-43 antibody that labels both mouse and human TDP-43 or total (ttl) TDP-43 (*red*), nuclear clearance of TDP-43 is obvious in both young and aged rNLS8 mice at 6 wks off Dox. **d** After 12 wks of transgene suppression, there is a return of endogenous mTDP-43 to the nucleus in young and aged mice. Scale bars = 100 μm. **e** Representative immunoblots showing RIPA-soluble hTDP-43∆NLS, mTDP-43 and GAPDH in rNLS8 SC before and after recovery compared to age-matched nTg control (cntrl) mice. There is no difference in the clearance of hTDP-43 or restoration of endogenous mouse TDP-43 between young and old animals. **f** Timeline of MN loss at L3–L5 levels of the SC in young (*red*) and aged (*blue*) rNLS8 mice showing that there is a precipitous decline in MN numbers while the transgene is expressed, which is completely halted by transgene suppression. Mean ± SD, *n* = 3–4 per time-point

Next, we assessed recovery from an ALS-like disease in aged rNLS8 mice. Following Dox-induced suppression of hTDP-43∆NLS after 6 wks of its expression, cytoplasmic hTDP-43 was cleared in both young and aged rNLS8 mice (compare Fig. 4a and b). In fact, the level of hTDP-43 expression was reduced by 86 and 77 % in young and aged mice respectively, after 12 wks of transgene suppression (Fig. 4e). Moreover, there was a return of endogenous mTDP-43 to the MN nucleus in both groups (compare Fig. 4c and d) to levels that are 75 and 93 % of the control mTDP-43 level (Fig. 4e). Therefore, there was no difference in hTDP-43 clearance or nuclear mTDP-43 restoration between young and old animals. Moreover, both young and old rNLS8 had a precipitous decline in MN numbers while the transgene was expressed, but in both groups this was completely halted by transgene suppression (Fig. 4f).

**Fig. 5:**
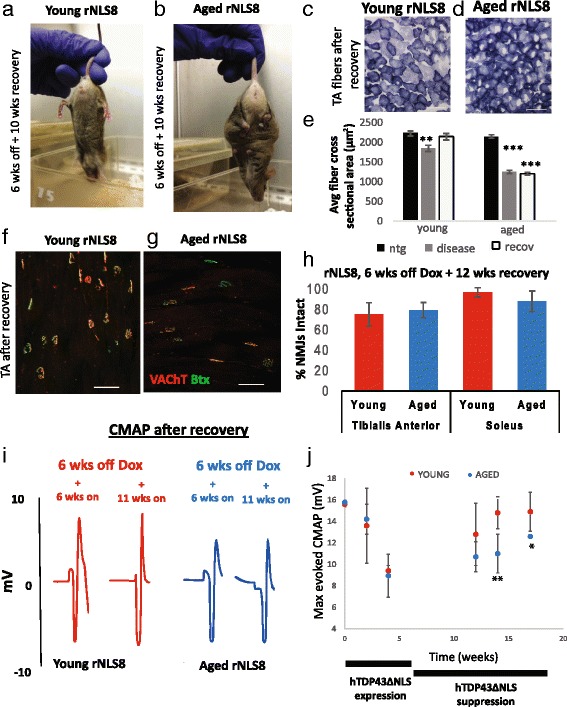
Suppressing the hTDP- 43ΔNLS transgene does not reverse muscle atrophy and dysfunction despite apparent muscle re-innervation in Tg aged mice. **a**-**b** Pictures of the phenotype of a representative young (**a**) and aged (**b**) rNLS8 mouse after 6 weeks of hTDP-43ΔNLS expression followed by 10 wk of suppression. Note that the young mouse splays its limbs normally, while the aged mouse is still clasping. **c**-**e** Representative pictures of muscle fibers from TAs of young (**c**) and aged (**d**) rNLS8 mice after 6 wks of transgene expression and 12 wks of subsequent suppression, stained with succinate dehydrogenase. Scale bars = 100 μm. **e** In both young and aged rNLS8 mice, average cross sectional fiber area in the TA muscle decreases during disease (6 wks off Dox, *grey bar*), reflecting muscle atrophy after denervation. However, in the young rNLS8 mice, after transgene suppression, the TA is re-innervated, and the average fiber size returns to baseline levels. Conversely, in the aged rNLS8 mice, despite apparent muscle re-innervation, the muscle remains significantly smaller than nTg controls. Mean ± SEM; **p < 0.01, ***p < 0.001. **f**-**h** After suppression of transgene expression for 12 wks, aged rNLS8 mice show the same level of re-innervation of TA and Sol muscles compared to young rNLS8 mice**. f**-**g**. Representative muscle cryosections from the TA of a young (**f**) and aged (**g**) rNLS8 mouse after long transgene suppression. Scale bars = 100 μm. **h** Quantification of intact NMJs in young (*red*) and aged (*blue*) TA and Sol after recovery, *n* = 3–4, mean ± SD. **i**-**j** Evoked CMAPs in the GC muscle after stimulation of the sciatic nerve in young (*red*) and aged (*blue*) rNLS8 mice that are back on Dox after 6 wks off show a more robust motor recovery in young animals. **i** Individual traces from the same animals at two different time-points showing the M-wave. **j** CMAP measurements significantly decrease with hTDP-43 expression, but can recover following transgene suppression and muscle re- innervation. Unlike young rNLS8 mice, the aged mice never return to their original, pre-disease baseline and have significantly lower maximum evoked CMAPs from the GC than young rNLS8 mice after 8 and 10 week of recovery. Data are mean ± SD, *n* = 3–7 animals per genotype, *p < 0.05, **p < 0.01

After having established that the cell bodies of MNs can recover from disease, we next investigated changes at the synapse and we observed that the aged mice did not appear as healthy as the young rNLS8 mice after transgene suppression. Young rNLS8 mice gained weight and began to splay their hind limbs normally after only a couple wks of Dox reintroduction (Fig. 5a). In contrast, most aged rNLS8 mice still exhibited symptoms after 10–12 wks of transgene suppression (Fig. 5b). Further, the TA muscles of aged rNLS8 mice remained atrophied after 12 wks of hTDP-43ΔNLS suppression, whereas young TA fibers returned to their pre-disease average cross-sectional size (Fig. 5c-e). The TA muscle remained significantly smaller than nTg controls in the aged rNLS8 mice, despite apparent muscle re-innervation at levels similar to young rNLS8 mice for both that muscle and the soleus, as assessed by overlap of vesicular acetylcholine transporter (VAChT) positive motor terminals and acetylcholine receptors indicating intact NMJs (Fig. 5f-h).

In addition to these morphological measures of muscle innervation, functional motor recovery was also assessed by measuring evoked compound muscle action potential (CMAP) in the GC muscle after stimulation of the sciatic nerve. Young and aged rNLS8 mice had repeated measurements taken at various time-points after the transgene was suppressed and the maximum peak-to-peak value of the early stimulus response (the M-wave, indicative of muscle contraction) increased over time after Dox reintroduction for both groups (Fig. 5i). However, unlike young rNLS8 mice, the aged mice never returned to their original, pre-disease baseline level (Fig. 5j). Therefore, despite a correction at the level of the MN cell body, suppressing the hTDP-43ΔNLS transgene did not reverse muscle atrophy and dysfunction despite apparent muscle re-innervation in aged mice.

**Fig. 6:**
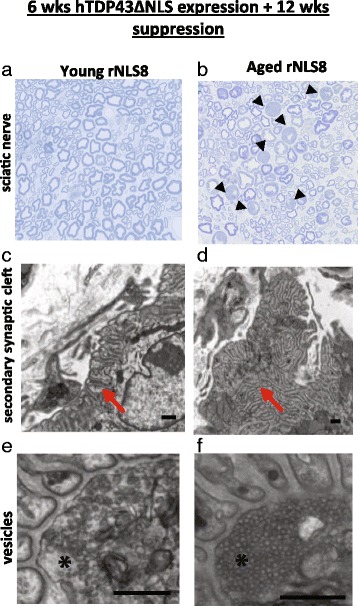
Functional motor recovery is likely diminished in rNLS8 mice because of impairments at the NMJ. **a**-**b** Representative pictures of sciatic nerves from a young (**a**) and aged (**b**) rNLS8 mice after 12 wks of transgene suppression, cut transversely at 1 μm and stained with toluidine blue and imaged by light microscopy at 40× to show axon morphology. The black arrowheads point to degenerating axons in the aged sciatic nerve section. **c**-**f** At the EM level, NMJ ultrastructural differences are apparent between the young and aged recovered rNLS8 mice. **c**-**d** While the TA of the young rNLS8 mouse still has junctional folds that are arranged in an organized way, mostly linearly, (see *red arrow* in **c**) the aged junctional folds are more often highly disorganized (see *red arrow* in **d**). Further, the NMJs of the young animal were more often completely re-innervated, whereas many of the junctional folds of the aged animal were only partially supplied by a nerve. **e**-**f** The synaptic vesicles were packed much more densely in the nerve terminal of the aged mouse (* in **f**) compared to the young mouse (* in **e**). Scale bars, 500 nm

To better understand the synaptic deficits observed in aged recovery animals, we looked more closely at the axons and NMJs of a young and an aged mouse after 6 wks of transgene expression followed by 12 wks of suppression and assessed qualitative differences between the two animals. We noted many more degenerated axons in the sciatic nerve of the aged rNLS8 animals (compare Fig. 6a and b). At the electron microscopic (EM) level, NMJ structural differences were also apparent between the recovering young and aged rNLS8 mice. First, the post-junctional folds of the motor endplates of the TA were often more disorganized in aged mice, whereas they remained more regular and linear in the young mouse (Fig. 6c-d). While both the young and old animal had some completely vacant endplates, of the ones that had a nerve terminal present, the old animal’s folds were much more often only partially re-innervated. Further, when the nerve terminals at the primary synaptic clefts were examined, the vesicles in the aged mice were packed much more tightly than in the young animal (Fig. 6e-f). Therefore, we speculate that the functional motor recovery is likely diminished in the aged rNLS8 mice because of these impairments at the NMJ.

## Discussion

In this study, we address a major unmet need in the field by modeling the interaction between ALS and aging, using a transgenic mouse in which hTDP-43∆NLS could be inducibly expressed to cause a build-up of TDP-43 in the cytoplasm, concurrent with the clearance of endogenous nuclear TDP-43. Unexpectedly, we did not find changes in the appearance of the neuromuscular disease in 12+ month old mice, even after repeated rounds of transgene cycling to stress MNs. Like in humans, where age-related MN loss has been reported beyond the seventh decade of life [28], we did find that our wild-type aged mice had minor MN loss associated with age (Fig. 1h), but this loss of MNs was not increased as a result of 4 wks of chronic hTDP-43∆NLS expression. It has also been reported that humans have an 18 % reduction in whole muscle cross-sectional area and 25 % fewer muscle fibers in older adults compared with young adults [19], but we did not see this change in our aged nTg mice (Fig. 5e) or in aged rNLS8 animals prior to transgene induction. This could be because we used mice that are middle-aged to better model ALS, which has most recently been reported to be diagnosed when patients are on average 60.7 years old (median: 61.5 years) [17]. Further, axonal dieback was not increased in our aged rNLS8 mice at 4 wks off Dox and the gross motor symptoms occurred at the same time as in the young animals (Fig. 1b-e).

Instead, we found that differences only emerged in the hind limb muscles and lumbar SC at later disease stages, at 6+ wks off Dox. It has been previously reported that aged rodents have an increased susceptibility to neuron loss following axonal damage within the retina [33], so our current report extends the results of the prior study now to spinal MNs. We also report that time to death following transgene induction is hastened by about 3.5 wks in aged rNLS8 mice (Fig. 3c). This is not surprising, as accelerated disease progression has been reported with increasing age for a wide range of disorders from HIV infection [26] to cardiovascular disease [24]. Our data in this study fit well with the observation that human ALS patients who are younger at the time of onset of symptoms have longer lengths of survival [4, 17, 23]. This could be because the diverse effects of aging in older subjects renders them more susceptible to adverse secondary effects of disease, rather than disease-specific primary events.

Perhaps our most important finding here is that aged rNLS8 mice are less capable of recovery from an ALS-like disease than younger mice. Though our older animals did improve when the transgene was suppressed, this recovery was slower and may never be functionally complete. Notably, given that the aged and young animals both reach the same level of hindlimb muscle innervation after 12 wks of transgene suppression (Fig. 5h) despite the aged rNLS8 mice having had significantly fewer intact NMJs at 6 wks off Dox (Fig. 3a), we assume that the aged animals had even more nerve regrowth over those 12 wks than young animals. However, when we looked specifically at these nerves (Fig. 6a-b), we saw that the organization of the aged peripheral sciatic nerves was consistent with more damaged and degenerating axons than the young nerves. Further, these aged nerves often failed to make complete connections with the post-junctional folds in the muscle. Therefore, what underlies recovery from motor neuron disease is both the regrowth of motor axons, and also their appropriate reconnection at the neuromuscular synapse. This observation is important to consider for the future design of clinical trials, wherein it will be important to compare the trajectories of individual trial subjects with age-matched patients, rather than the entire group.

When we observed that the aged rNLS8 mice still exhibited motor impairments after the hTDP- 43ΔNLS transgene was suppressed, we imagined several possible explanations for the incomplete recovery, centered on deficits at the MN cell body, the axon, or the NMJ. First, we verified that aged MNs were able to clear cytoplasmic hTDP-43 and that nuclear TDP-43 could be restored. We found that both of these events occurred in a similar manner as in the young rNLS8 MNs (Fig. 4). Next, because many previous studies have demonstrated that the axons of aged rodents have decreased regenerative capabilities [31] and reached their targets at a much slower rate [5, 13, 22, 30], we verified that the nerve terminals reached the muscles. By looking at individual muscles at low power and quantifying VAChT-positive nerve terminals that had reached motor end-plates (Fig. 5g–h), we found that there was indeed as much overlap as in young rNLS8 animals, indicating that NMJs reformed with axon re-growth and collateral sprouting. Finally, we examined the ultrastructure of these reformed NMJs by EM to potentially understand the aged animals’ diminished CMAP, continued hind limb clasping, and muscle fiber atrophy. A qualitative analysis of an aged versus a young TA muscle after 12 wks of transgene suppression showed interesting structural alterations in the NMJs of the aged rNLS8 mouse. Broadly, the systems of both primary junctional folds and secondary clefts appeared to be much more disorganized in the aged animal. Further, we observed a decreased nerve terminal area and post-synaptic folds devoid of nerve terminals in the aged muscle, with those denervated portions often without Schwann cell coverage. We also saw a marked difference in the numbers and densities of synaptic vesicles, with the aged animal having a higher density of vesicles (Fig. 6f), which is in contrast to a previous study reporting lower numbers of vesicles in aging albeit in a very old (34 month) mouse [3].

Our findings now add to several other studies that have reported defective NMJ re-establishment in older animals [2, 9, 15], though ours differ from these because our experimental denervation does not rely on a transection or crush of the peripheral nerve. Instead, what we modeled here is axonal dieback from the muscle as a result of pathological processes occurring in the MN cell body due to aggregation and mislocalization of TDP-43, and the effects of aging on this process as well as the recovery from it. Like the studies with nerve injury before, we find that a key feature of the re-innervation of aged muscle is excitation–contraction uncoupling, which causes a failure to restore muscle mass and strength [11]. A large body of current ALS research focuses on pathological processes affecting the spinal MN cell body, but it is clear in this study that some of the most key differences that impact the overall health and motor functioning of the aged rNLS8 mice are happening at the NMJ. Future studies will examine whether the surviving MNs have a lessened capacity to meet new, increased bioenergetic demands.

## Conclusions

In summary, we have extended our previous work on rNLS8 mice from merely modeling the pathology and motor symptoms associated with sporadic ALS [27, 32], to incorporating the temporal component of the disease as well. We believe that these studies will be an important basis for the future design and evaluation of therapies designed to slow denervation and promote re-innervation in middle-aged ALS patients.
